# The Role of Estrogen Receptor β in the Dorsal Raphe Nucleus on the Expression of Female Sexual Behavior in C57BL/6J Mice

**DOI:** 10.3389/fendo.2018.00243

**Published:** 2018-05-16

**Authors:** Kazuhiro Sano, Chihiro Morimoto, Mariko Nataka, Sergei Musatov, Mumeko C. Tsuda, Naoko Yamaguchi, Toshiro Sakamoto, Sonoko Ogawa

**Affiliations:** ^1^Laboratory of Behavioral Neuroendocrinology, University of Tsukuba, Tsukuba, Japan; ^2^Laboratory of Molecular Neurosurgery, Department of Neurological Surgery, Weill Cornell Medical College, New York, NY, United States; ^3^Department of Medicine, Aichi Medical University, Nagakute, Japan; ^4^Department of Health Sciences, Kyoto Tachibana University, Kyoto, Japan

**Keywords:** adeno-associated virus-mediated RNA interference, estrogen receptor β, estrogen receptor α, lordosis, dorsal raphe nucleus, estrogen, progesterone, serotonin

## Abstract

17β-Estradiol (E_2_) regulates the expression of female sexual behavior by acting through estrogen receptor (ER) α and β. Previously, we have shown that ERβ knockout female mice maintain high level of lordosis expression on the day after behavioral estrus when wild-type mice show a clear decline of the behavior, suggesting ERβ may be involved in inhibitory regulation of lordosis. However, it is not identified yet in which brain region(s) ERβ may mediate an inhibitory action of E_2_. In this study, we have focused on the dorsal raphe nucleus (DRN) that expresses ERβ in higher density than ERα. We site specifically knocked down ERβ in the DRN in ovariectomized mice with virally mediated RNA interference method. All mice were tested weekly for a total of 3 weeks for their lordosis expression against a stud male in two consecutive days: day 1 with the hormonal condition mimicking the day of behavioral estrus, and day 2 under the hormonal condition mimicking the day after behavioral estrus. We found that the level of lordosis expression in ERβ knockdown (βERKD) mice was not different from that of control mice on day 1. However, βERKD mice continuously showed elevated levels of lordosis behavior on day 2 tests, whereas control mice showed a clear decline of the behavior on day 2. These results suggest that the expression of ERβ in the DRN may be involved in the inhibitory regulation of sexual behavior on the day after behavioral estrus in cycling female mice.

## Introduction

The expression of female sexual behavior undergoes cyclic change during an estrus cycle. An ovarian hormone, 17β-estradiol (E_2_) plays an essential role in this cyclic regulation of the behavior. In rodents, lordosis, a stereotypical female sexual behavior is expressed at high levels only during the time around ovulation, the period called as “behavioral estrus” occurring subsequently to the exponential elevation of circulating E_2_. E_2_ is known to act through at least two receptor subtypes, estrogen receptor (ER) α and β. Studies using knockout mouse models have shown that the level of sexual behavioral expression is greatly reduced in ERα knockout (αERKO) females (1–3) whereas in the ERβ knockouts (βERKO), such alteration is not observed (4, 5). It has also been reported that the selective ERα agonist, propyl-pyrazole triol facilitates female sexual behavior in ovariectomized female rat whereas the selective ERβ agonist, diarylpropionitrile (DPN) is unable to do so (6). Furthermore, Musatov et al. (7) demonstrated that the site-specific knockdown of ERα in the ventromedial nucleus of the hypothalamus (VMN) leads to a complete disappearance of female sexual behavior in mice. These studies collectively suggest that E_2_ action through ERα, particularly in the VMN, is necessary for normal display of female sexual behavior whereas ERβ may not be involved in facilitatory regulation of female sexual behavior by E_2_. However, close observation of gonadally intact βERKO mice has revealed an intriguing behavioral phenotype that the period of behavioral estrus may be extended in βERKO females (4). In these mice, the level of sexual receptivity was reported to stay high until the day after behavioral estrus when their respective wild-type (WT) mice show a clear decline of the behavior. In addition, except the day of behavioral estrus and the day after, βERKO females express very little receptive behavior similar to WT females (4). To replicate this finding in more hormonally controlled setting, we performed a preliminary experiment in ovariectomized βERKO females treated with exogenous ovarian steroids. We found that these females continuously showed elevated levels of lordosis expression 72 h after estradiol benzoate (EB) and 24 h after progesterone administration that mimic typical hormonal condition of the day after behavioral estrus (unpublished data). These findings collectively suggest that E_2_ is not only promoting sexual behavior in females during the behavioral estrus with ERα dependent mechanisms but also involved in active inhibition of lordosis expression on the day after behavioral estrus by acting through ERβ. To further test this hypothesis and elucidate possible neural mechanisms of ERβ-mediated inhibitory regulation of lordosis, we aimed to identify the responsible brain site(s) in this study.

The dorsal raphe nucleus (DRN) is one of the brain areas that possibly act inhibitory on the expression of female sexual behavior. Lesions of this area are reported to increase lordosis expression in both EB-treated ovariectomized female rat and EB-treated castrated male rat (8–11) whereas the electrical stimulation of the DRN can lead to marked and immediate suppression of lordosis in ovariectomized female rat hormonally primed with EB and progesterone (12). In addition to its possible inhibitory role in the regulation of female sexual behavior, the DRN is also known for high levels of ERβ expression that is about two-fold of that of ERα within the nucleus (13, 14). Thus, it is reasonable to hypothesize that the DRN may be the brain site responsible for ERβ-mediated inhibitory action of E_2_ in female sexual behavior. To test this hypothesis, we employed a virally mediated RNA interference method and examined the effects of site-specific knockdown of ERβ in the DRN on the expression of lordosis behavior.

## Materials and Methods

### Subjects

Adult C57BL/6J female mice originally purchased from a commercial breeder (CLEA, Japan) and maintained in a breeding colony at the University of Tsukuba. All mice were housed under standard conditions (23 ± 2°C) with a 12:12-h light/dark cycle (lights off at 12:00). Food and water were provided *ad libitum*. All procedures were approved by the Animal Care and Use Committee and the Recombinant DNA Use Committee at the University of Tsukuba and conducted strictly in accordance with the National Institutes of Health guidelines. All efforts were made to minimize the number of animals and their suffering.

### Design of Small Hairpin (sh) RNA for ER β Silencing

Adeno-associated virus (AAV) vectors expressing an shRNA against either the sequence specific for the ERβ gene (AAV-shERβ: 5′-GATCCCCGCCACGAATCAGTGTACCATCTTCCTGTCAATGGTACACTGATTCGTGGCTTTTTTGGAAT-3′ and 5′-CTAGATTCCAAAAAAGCCACGAATCAGTGTACCATTGACAGGAAGATGGTACACTGATTCGTGGCGGG-3′) or the sequence specific for luciferase (LUC) as control (AAV-shLUC: 5′-GATCCCCCCGCTGGAGAGCAACTGCATCTTCCTGTCAATGCAGTTGCTCTCCAGCGGTTTTTGGAAT-3′ and 5′-CTAGATTCCAAAAACCGCTGGAGAGCAACTGCATTGACAGGAAGATGCAGTTGCTCTCCAGCGGGGG-3′) were used. The nucleotides specific for ERβ or LUC are underlined. These vectors also express enhanced green fluorescent protein (GFP) as a reporter to visually detect transduced neurons.

### Stereotaxic Surgery

Adult female mice (11.4 ± 2.2 weeks old) were assigned to either vector treatment (i) AAV-shERβ [ERβ knockdown (βERKD)] or (ii) AAV-shLUC (Control). They were anesthetized with sodium pentobarbital (60 mg/kg) and placed in a stereotaxic frame. A 5 µl Hamilton syringe inclined at 25° was aimed at the DRN [anteroposterior, −4.96 mm, mediolateral, 0.00 mm; dorsoventral, −3.40 mm] that was determined based on The Mouse Brain Stereotaxic Coordinates (15). Each mouse was injected with 0.5 µl of either AAV-shERβ or AAV-shLUC (10^12^ packaged genomic particles) using a micropump injector (World Precision Instruments Inc., USA). The injection lasted 5 min, and the needle was left in place for an additional 10 min following the end of infusion. Mice were then group housed with their littermates (4–5 mice/cage) until they were used for behavioral studies.

### Ovariectomy and Hormone Treatment

Two weeks after stereotaxic surgery, all mice were ovariectomized (OVX) under isoflurane inhalation anesthesia. Mice were then individually housed in plastic cages (19 cm × 29 cm × 19 cm). All female mice were hormonally treated with weekly subcutaneous injections of EB (5 μg/0.1 ml sesame oil) followed by progesterone (P; 250 μg/0.1 ml sesame oil) at 44–46 h later, to mimic hormonal conditions in cycling females.

### Sexual Behavior Test

**Figure 1:**
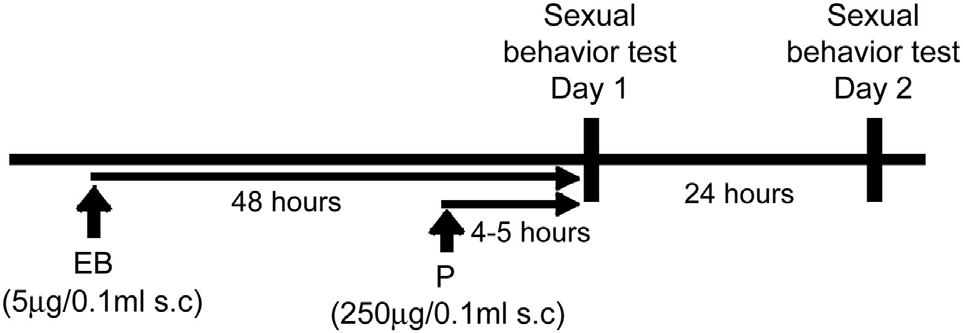
Experimental paradigm.

Starting 1 week after OVX, all mice were weekly tested for sexual behavior against a sexually experienced male (gonadally intact ICR/Jcl) mouse in the males’ home cages on two consecutive days, for a total of six trials during 3 weeks. For each week, mice were first tested 4–5 h after P injection which mimic the day of behavioral estrous (day 1), then tested again 24 h later (day 2) (Figure 1). Each test was performed during the dark phase (starting 2–3 h after lights off) of the light/dark cycle under red light illumination. Each test lasted until females received 15 mounts or intromissions. Male intromissions were terminated (after about 8 thrusts) by the experimenter after female’s behavioral responses were scored. Lordosis quotient (LQ) was calculated by dividing the number of lordosis responses by 15, the number of mounts or intromissions. In addition, each lordosis posture was given a score of 1–3 depending on the degree of dorsiflexion of the vertebral column and behaviors. For calculation of Lordosis Quality Scores, only lordosis responses that were given a score between 1 and 3 were included and averaged in each mouse. Mice with no lordosis response were excluded from the analysis in each test.

### Tissue Preparation for Immunohistochemistry

Two weeks after the completion of behavioral testing, all mice were given subcutaneous injection of EB (5 μg/0.1 ml sesame oil). Forty-eight hours later, the time point matching the hormonal condition of day 1 tests, they were deeply anesthetized with a solution of 1:1 mixture of sodium pentobarbital (60 mg/kg) and heparin (1,000 U/kg) and transcardially perfused with 0.1 M phosphate-buffered saline (PBS; pH 7.2) followed by 4% paraformaldehyde (PFA) in 0.1 M phosphate buffer (PB; pH 7.2). Brains were removed and post-fixed overnight at 4°C in 4% PFA in 0.1 M PB. They were then rinsed with 0.1 M PB and cryoprotected in 0.1 M PB containing 30% sucrose.

### Immunohistochemistry

Free-floating coronal sections (35 µm thickness) prepared on freezing microtome were split into four series (140 µm apart). One series of sections from each mouse was incubated in a rinse buffer, PBS-X (PBS and 0.2% Triton X-100), containing 1% hydrogen peroxide for 20 min to inhibit endogenous peroxidase activity, and then blocked in an incubation buffer (3% skim milk and 3% bovine serum albumin in PBS-X) for 2 h at room temperature. After the blocking process, they were incubated in a goat polyclonal anti-GFP antiserum (1:5,000; ab6673, Abcam) dissolved in the incubation buffer overnight at 4°C. Sections were then treated with a 1:250 dilution of biotinylated rabbit anti-goat secondary antibody (Vector Laboratories) in the incubation buffer for 2 h at room temperature, followed by 1 h incubation with avidin-biotin complex (Vectastain ABC Elite kit, Vector Laboratories) in TBS. After the completion of antiserum reaction procedures, sections were visualized with 0.02% diaminobenzidine and 0.003% hydrogen peroxide in TBS. A few sections from each group were also processed for immunohistochemical staining for ERβ. They were incubated in a rinse buffer, PBS-X, containing 1% hydrogen peroxide for 20 min, and then blocked in an incubation buffer (5% bovine serum albumin in PBS-X) for 2 h at room temperature. After the blocking process, they were incubated in a goat polyclonal anti-ERβ antiserum (1:1,000; Z8P, lot 10766190, Zymed Laboratories) dissolved in the incubation buffer for three days at 4°C. Sections were then treated with a1:250 dilution of biotinylated goat anti-rabbit secondary antibody (Vector Laboratories) in the incubation buffer for 4 h at room temperature, followed by 1 h incubation with avidin-biotin complex (Vectastain ABC Elite kit, Vector Laboratories) in TBS. After the completion of antiserum reaction procedures, sections were visualized with 0.03% diaminobenzidine, 0.15% NiNH_4_SO_4_, and 0.003% hydrogen peroxide in TBS.

All sections were mounted in gelatin-coated slides, air-dried and dehydrated through ascending alcohol series, which were cleared with xylene, and cover slipped with Permount (Fisher Scientific, USA). To verify the specificity of the immunohistochemical procedures, we included negative controls in which the primary antiserums were omitted from the staining procedure. In these conditions, neither cells nor fibers were stained.

The seven sections containing the DRN [Bregma −4.24 to −5.20 mm (15)] were obtained and photographed at 40× magnification with a digital camera mounted on an Olympus microscope (DP21, Olympus, Japan). To verify successful infusion of shRNA in the DRN, the distribution of GFP immunoreactive cells was evaluated on these seven sections.

### Statistics

Lordosis quotients in the Control and βERKD groups were analyzed by a three-way analysis of variance (ANOVA) for repeated measurements for the main effects of vector treatment, day, week, and their interactions. LQs in the missed βERKD group were analyzed by a two-way ANOVA for repeated measurement for the main effect of day and week, and their interactions. ANOVAs were followed by Bonferroni *post hoc* test when it was appropriate. Lordosis Quality Scores were analyzed and compared by Mann–Whitney *U* test between day 1 and day 2 in each week in each vector treatment group. All data were presented as mean ± SEM. Statistically significant differences were considered at *p* < 0.05 (two-tailed). LQs were analyzed using SPSS 21.0 (SPSS Inc., Chicago, IL, USA) statistical package, and Lordosis Quality Scores were analyzed using StatView 5.0.1 (SAS Institute Inc., Cary, NC, USA).

## Results

### Verification of Transfection

**Figure 2:**
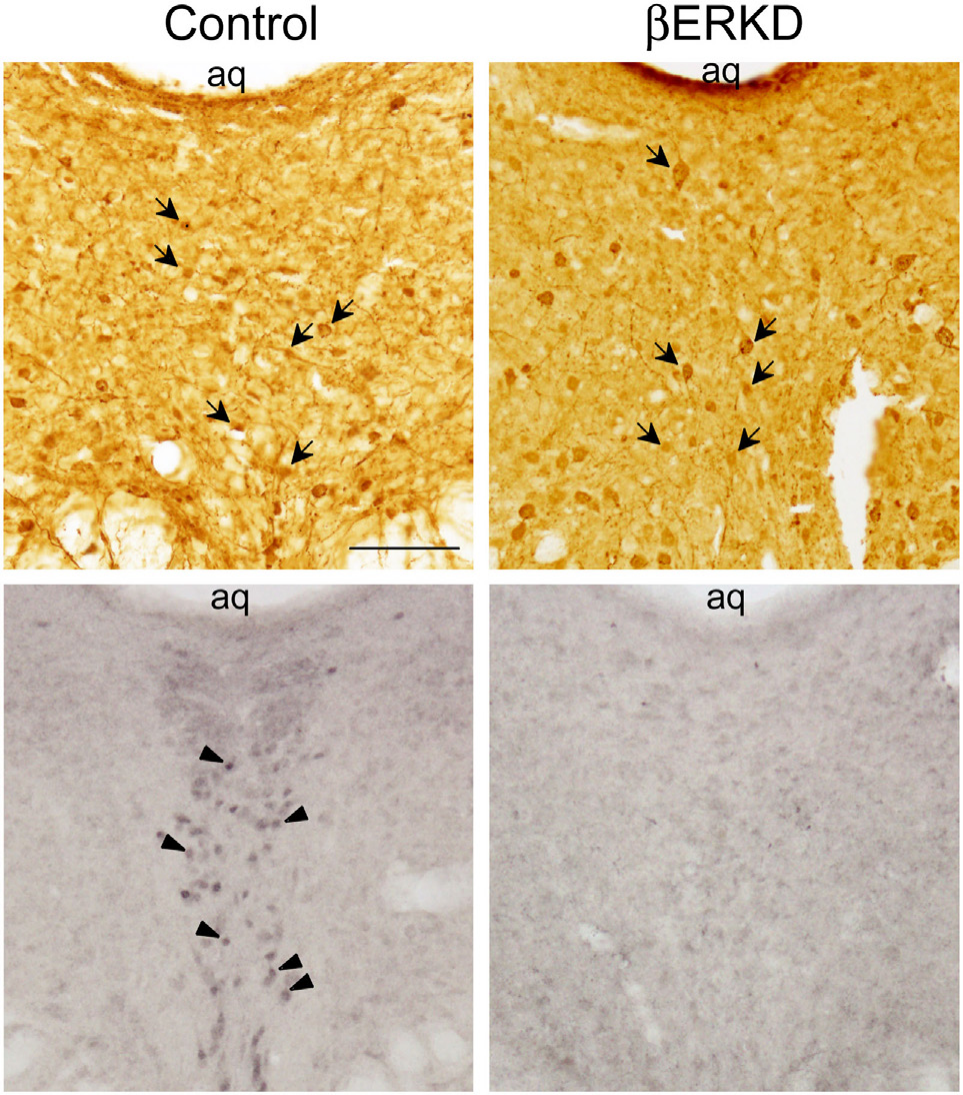
Representative photomicrographs of immunohistochemically labeled cells for green fluorescent protein (GFP) in brain sections of control and ERβ knockdown (βERKD) groups (top panels), and those for estrogen receptor (ER) β in the adjacent sections (bottom panels). GFP and ERβ immunoreactive cells were detected by the methods described in the present and previous studies (16). The scale bar in the panel represents 100 µm. Arrows in the top panels indicate GFP stained cells, and arrow heads in the bottom panel indicate ERβ stained cells.

Similar to previous reports in the medial amygdala and medial preoptic area (16), the efficacy of the vector in silencing the expression of ERβ site-specifically in the DRN was confirmed as shown in Figure 2.

**Figure 3:**
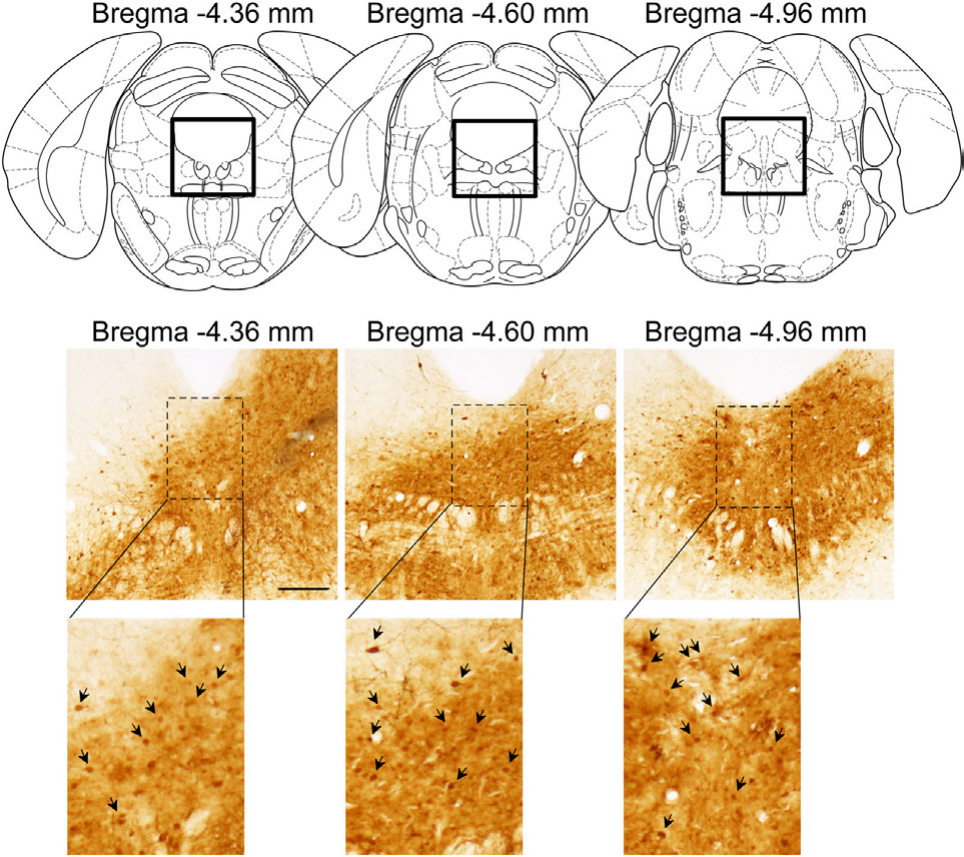
Representative distribution pattern of adeno-associated virus within the dorsal raphe nucleus determined based on green fluorescent protein immunohistochemistry. The scale bar represents 200 µm.

**Figure 4:**
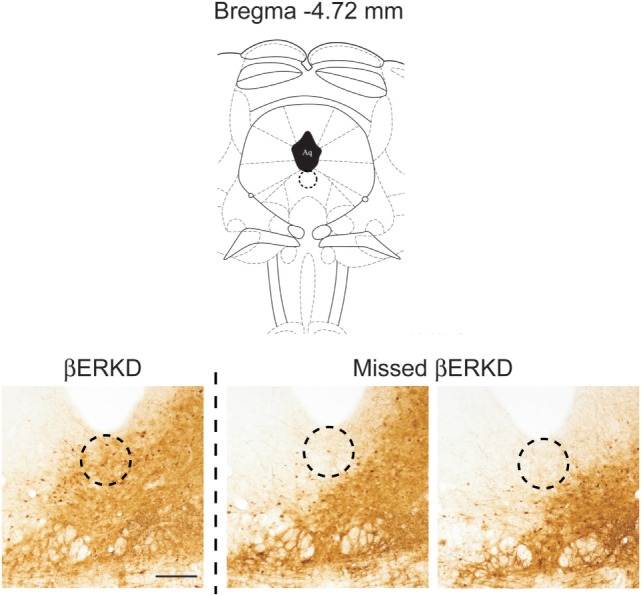
Comparison of distribution pattern of adeno-associated virus within the mediodorsal area of dorsal part of the dorsal raphe nucleus (Bregma −4.72 mm) determined by green fluorescent protein immunohistochemistry in successful ERβ knockdown (βERKD) (left) and missed βERKD (middle and right) mice. The scale bar represents 200 µm.

Successful infusion of shRNA in the DRN was confirmed in 12 females injected with AAV-shLUC (Control) and 10 females with AAV-shERβ (βERKD) (Figure 3). In addition to these mice in which AAV-shERβ was successfully infused into the entire DRN, there were five females in which AAV-shERβ was not spread in the mediodorsal area of dorsal part of the nucleus (Figure 4). These animals were classified as a missed βERKD group, and their behavioral data were analyzed separately. The mediodorsal area of dorsal part of the DRN was defined as the area within a 0.2 mm diameter circle placed on right below the bottom edge of the aqueduct and ranging from Bregma −4.48 to −4.84 mm (15) (Figure 4).

### Effect of ERβ Knockdown in the DRN on the LQ

**Figure 5:**
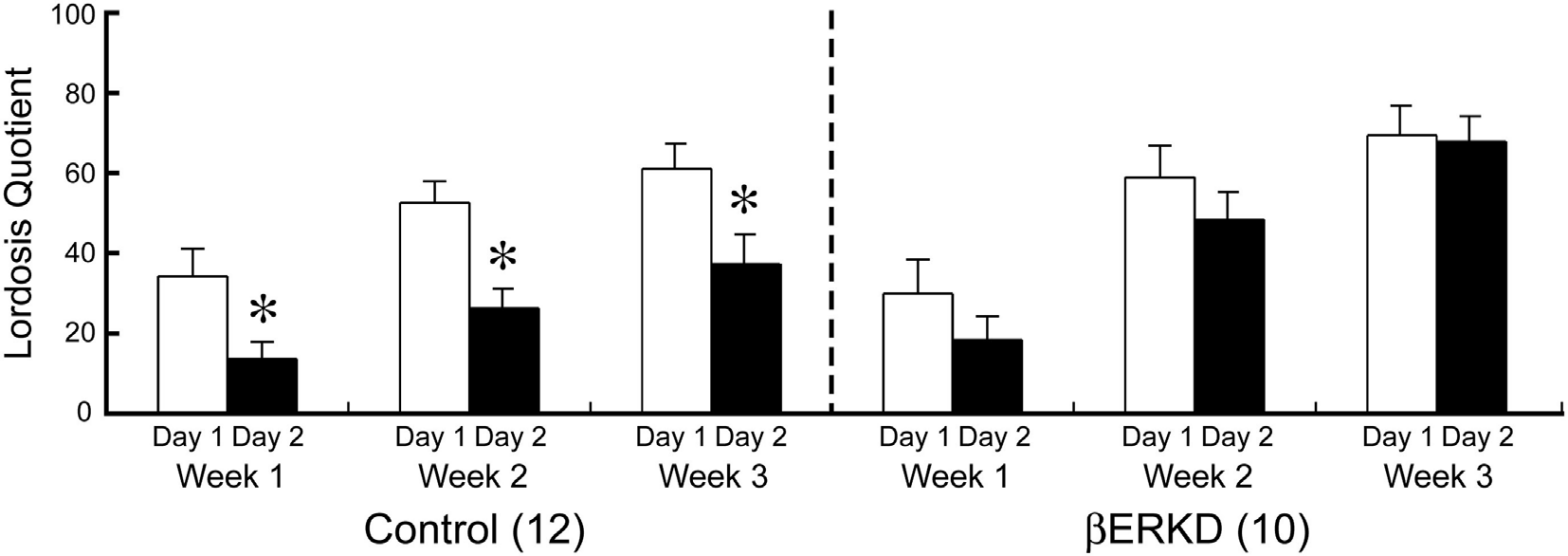
Effect of estrogen receptor (ER) β knockdown in the dorsal raphe nucleus on Lordosis quotient (LQ). LQ of control (left) and ERβ knockdown (βERKD) (right) in each test. *P < 0.05 vs. day 1. Data presented as mean ± SEM.

Knocking down of ERβ in the DRN dramatically affected LQ in day 2, but not in day 1. As shown in Figure 5, Control and βERKD showed similar levels of LQ on day 1 test in each week. In the Control group, the LQs were greatly decreased on day 2 compared with day 1 in all three weekly tests, whereas βERKD showed similar levels of LQ on day 2 as those on day 1 in all three tests. Three-way repeated measures ANOVA revealed significant effects of Day [*F*(1, 20) = 22.247, *P* < 0.0001], Treatment [*F*(1, 20) = 4.881, *P* < 0.05], and Week [*F*(2, 40) = 37.487, *P* < 0.0001]. There were significant interactions between Treatment and Day [*F*(1, 20) = 5.518, *P* < 0.05], Treatment and Week [*F*(2, 40) = 3.876, *P* < 0.05]. There were no statistically significant interactions between Day and Week [*F*(2, 40) = 0.390, ns] or Treatment, Day, and Week [*F*(2, 40) = 0.381, ns]. *Post hoc* analysis revealed that LQ on day 2 was lower than day 1 in the Control group alone (the day 1 vs. the day 2 in each test, *P* < 0.01, for control) in each test. However, there was no significant difference between day 1 and day 2 in LQ of βERKD in each test.

### Effect of ERβ Knockdown in the DRN on the Lordosis Quality Score

**Figure 6:**
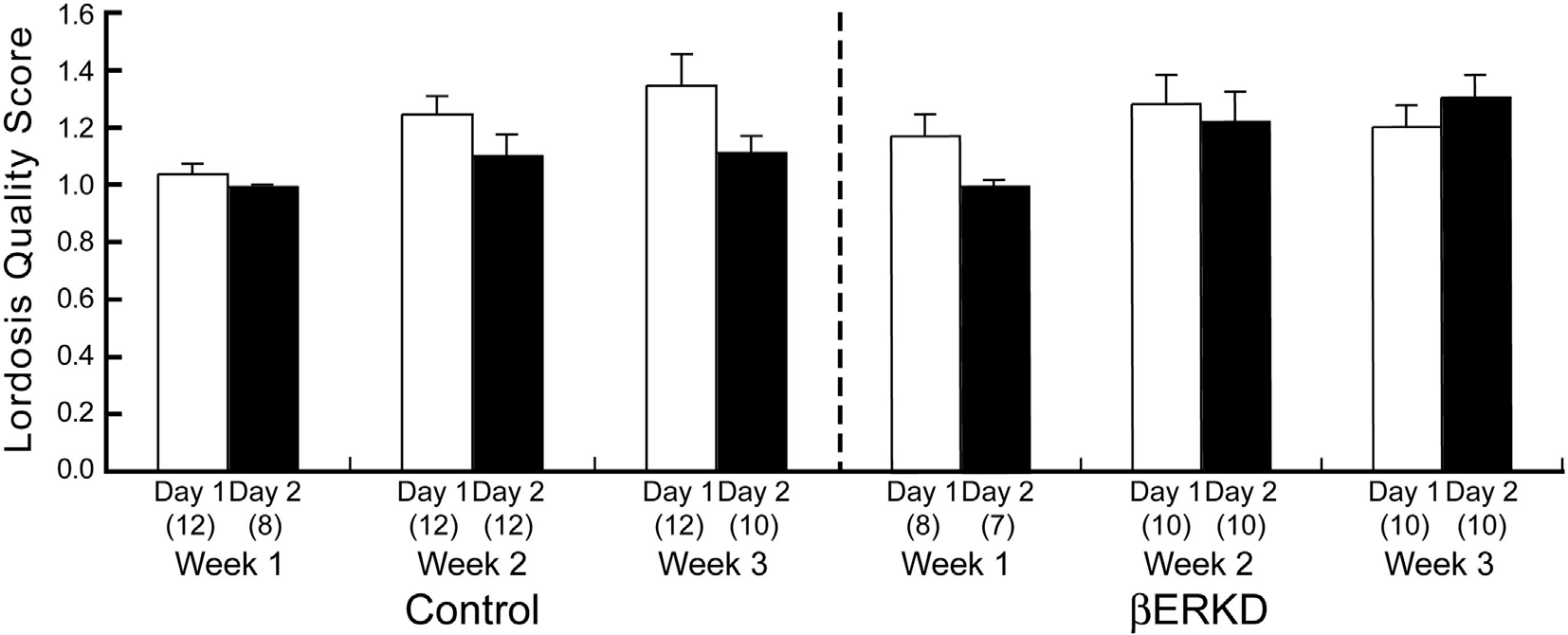
Lordosis Quality Score of control (left) and ERβ knockouts (right) mice in each test. There was no difference between day 1 and day 2 in each week regardless of treatment group. The number in parentheses showed the number of mice which expressed lordosis (i.e., lordosis score of higher than 1) in each test.

Estrogen receptor β knockdown did not affect the Lordosis Quality Score (Figure 6). Mann–Whitney *U* test revealed that there was no difference between day 1 and day 2 of Lordosis Quality Score in each week regardless of treatment group (Control: Week 1; *U* = 44.000, Week 2; *U* = 40.500, Week 3; *U* = 33.500, βERKD: Week 1; *U* = 16.000, Week 2; *U* = 43.000, Week 3; *U* = 33.000, all ns).

### LQ of Missed βERKD Group

Behavioral data shown above included only mice in which the range of vector infusion covered the entire targeted area, but not elsewhere. These βERKD mice maintained the LQ that was equivalent to day 1 in day 2. On the other hand, such effect was absent if the virus did not spread into the mediodorsal area of dorsal part in the DRN. Two-way repeated measures ANOVA revealed significant effects of Day [*F*(1, 4) = 21.574, *P* < 0.05] and Week [*F*(2, 8) = 7.704, *P* < 0.05]. There were no statistically significant interactions between Day and Week [*F*(2, 8) = 0.141, ns].

## Discussion

In this study, we demonstrated that knocking down of ERβ in the DRN altered female sexual behavior on the day after behavioral estrus without affecting the behavior on the day of behavioral estrus. Our results showed that LQ between βERKD and Control mice was at a similar level on day 1 test performed at 44–46 h after EB and 4–6 h after progesterone injection that mimicked hormonal states of the day of behavioral estrus. By contrast, on day 2 test which corresponds to the day after behavioral estrus, βERKD mice still showed similar levels of LQ in comparison with their LQ on day 1 in each of three test. Control mice showed greatly decreased levels of LQ on day 2 compared with day 1 (Figure 5). These findings are consistent with previous studies using βERKO mice in which both gonadally intact and hormonally manipulated ovariectomized βERKO females continuously showed elevated levels of receptivity on the day after behavioral estrus [(4); unpublished data]. Thus, our finding suggests that the DRN is one of the brain sites responsible for the ERβ-mediated inhibitory regulation of female sexual behavioral expression on the day after behavioral estrus. On the other hand, Lordosis Quality Score that indicates the quality of each lordosis was not different between day 1 and day 2 in both βERKD and Control groups (Figure 6). The brain sites responsible for the qualitative aspect of lordosis posture and possible involvement of gonadal steroids are not known. Our data indicate that ERβ expressed in the DRN may not be involved in the qualitative regulation of lordosis responses.

**Figure 7:**
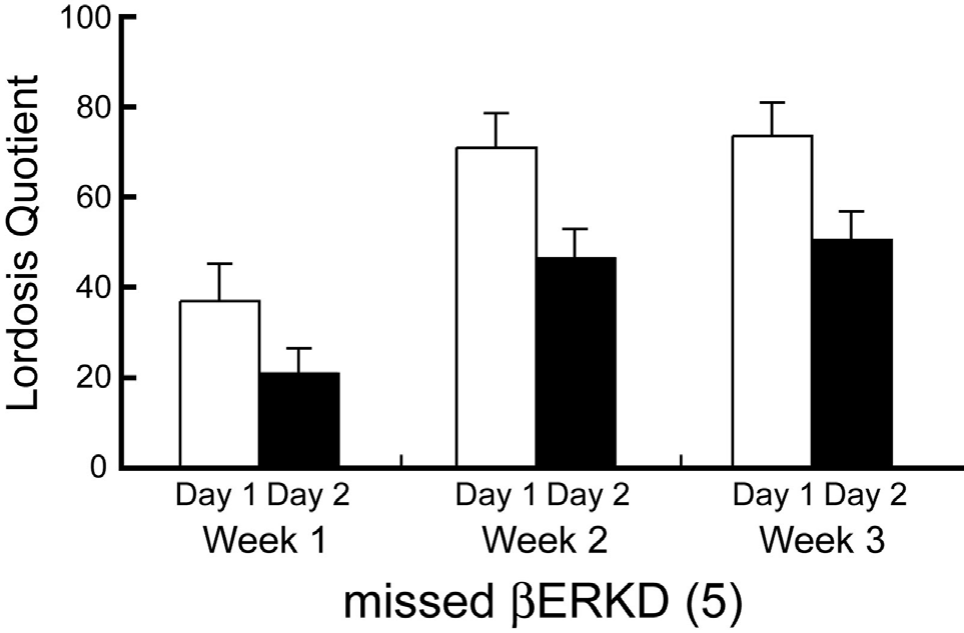
Lordosis quotient of missed ERβ knockdown (βERKD) mice. Data presented as mean ± SEM.

Furthermore, the knockdown effect was absent if the virus did not spread into the mediodorsal area within the dorsal part of the DRN even though the infusion was successful elsewhere within the nucleus (Figures 4 and 7). This finding may indicate that even within the DRN, the mediodorsal area of dorsal part is the most critical area for the ERβ-mediated inhibition of female sexual behavior on the day after behavioral estrus.

The exact mechanism of this ERβ-mediated inhibitory regulation of female sexual behavior has yet to be elucidated. However, it is possible to hypothesize that ERβ in the DRN may be involved in inhibition of female sexual behavior by intervening the serotonergic systems. Serotonin is known to act inhibitory on the expression of female sexual behavior through its binding to 5HT-1A receptors (17). The DRN is the largest serotonin synthesizing nucleus whose projections directly innervate the VMN and MPOA where 5HT-1A receptors are highly abundant (18–20). Selective lesions of serotonergic neurons projecting from the DRN to the VMN have been reported to facilitate lordosis (21). On the other hand, site-specific activation of 5HT-1A receptors either in the VMN or MPOA by local infusion of selective agonist, 8-hydroxy-2-(di-*n*-propylamino)tetralin (8-OH-DPAT) reduces the expression of lordosis in hormonally primed ovariectomized and gonadally intact proestrus female rats (22–24). Within the DRN, more than 95% of ERβ expressing neurons are serotonergic (14), and the majority of serotonergic neurons that project into the MPOA express ERβ (25). Furthermore, the administration of 17β-estradiol or ERβ-specific agonists, DPN to ovariectomized female rats are reported to enhance mRNA expression of tryptophan hydroxylase 2 (TPH 2), the rate-limiting enzyme for serotonin synthesis (26). In addition, the TPH 2 mRNA expression is greatly reduced in the DRN of βERKO female mice (14). Thus, ERβ in the DRN is possibly mediating the inhibition of female sexual behavior by upregulating the activity of the serotonergic system within the DRN that further activates 5HT-1A receptors expressed in hypothalamic regions such as the VMN and MPOA. The expression of 5HT-1A receptors in these regions seems to be also upregulated by estrogen (20, 27). Interestingly, a recent study by Spiteri et al. (28) reported that the site-specific knockdown of ERα in the MPOA, the region also known for its inhibitory role on the expression of female sexual behavior, increase the lordosis expression in response to a low dose EB treatment that generally fails to stimulate lordosis in ovariectomized females. Thus, the inhibitory action of the MPOA on the female sexual behavior seems to be ERα mediate although the crucial role of ERα in the VMN in the facilitation of the behavior is well known (7).

Collectively with these studies, our present findings provide a significant piece to lay out the possible mechanism underling the cyclic regulation of female sexual behavior. Thus, on the day of behavioral estrus, high levels of circulating estradiol simultaneously (1) facilitate behavioral expression *via* ERα-mediated genomic action in the VMN, (2) upregulate 5HT-1A receptor expression *via* ERα in the hypothalamic areas such as the VMN and MPOA, while (3) upregulate TPH 2, the rate-limiting enzyme for serotonin synthesis in the DRN *via* ERβ. On the day after behavioral estrus, serotonin release by serotonergic neurons in the DRN may activate 5HT-1A receptors in the VMN and/or the MPOA and inhibit lordosis expression.

In addition to serotonin, progesterone is also known to inhibit the expression of female sexual behavior in its long-term effect while its short-term effect is facilitatory. Progesterone acts through progesterone receptor (PR), and the expression of the receptor is highly dependent on estrogen action. In female mice, the expression of PR in the VMN is known to be upregulated by estrogen *via* ERα (7). Whereas in the DRN, it seems to be ERβ dependent since EB administration still upregulate PR expression in the DRN of ovariectomized αERKO female mice (29). Moreover, estrogen-induced PR expression in the DRN found in αERKO female is suspected to be on the serotonergic neuron since the majority of PR immunoreactive cells coexpressed TPH immunoreactivity in these females (29). Therefore, it is reasonable to hypothesize that PR upregulation by E_2_ in the DRN is mediated ERβ by and this progesterone–PR signaling pathway, in turn, may contribute the activation of serotonergic system that inhibits the expression of female sexual behavior on the day after behavioral estrus.

In this study, we focused on lordosis that reflects more of consummatory aspects. However, we cannot exclude the possible effect of ERβ knockdown in the DRN on behavioral components other than lordosis itself. Ogawa et al. (4) reported that gonadally intact βERKO female mice exhibited slightly higher levels of proceptivity throughout the estrous cycle compared with WT mice. Thus, in future experiments, it will be important to investigate the site-specific involvement of ERβ in the DRN in the appetitive aspects of female sexual behavior. It is also important to directly monitor or manipulate neuronal activity of ERβ- and/or PR-expressing cells and the serotonergic system within the DRN during both estrus and non-estrus phase to further elucidate neuronal and intracellular mechanisms of ERβ-mediated inhibitory regulation of female sexual behavior. Moreover, it is our great interest to investigate site-specific involvement of ERβ in brain regions other than the DRN such as the MPOA, the region also involved in the inhibitory regulation of female sexual behavior and known to highly express ERβ.

## Conclusion

The main aim for this study was to elucidate site-specific involvement of ERβ expressed in the DRN in the inhibitory regulation of sexual behavior in female mice. This study demonstrated that βERKD mice continuously showed elevated levels of receptivity even on the day after behavioral estrus, whereas control mice showed greatly reduced receptivity on the day after behavioral estrus compared with the day of estrus. Therefore, the findings in this study suggest that ERβ in the DRN may be involved in the inhibition of female sexual behavior on the day after behavioral estrus. This is the first study to demonstrate the site-specific involvement of ERβ in the inhibitory regulation of female sexual behavior.

## Ethics Statement

All procedures were approved by the Animal Care and Use Committee and the Recombinant DNA Use Committee at the University of Tsukuba and conducted strictly in accordance with the National Institutes of Health guidelines. All efforts were made to minimize the number of animals and their suffering.

## Author Contributions

KS, CM, and SO designed research and wrote the paper. KS, CM, MN, SM, MT, NY, and TS performed research and analyzed data. SM and SO contributed new reagents/analytic tools; KS and CM equally contributed to the study. SM designed and prepared the viral vectors expressing shRNAs used in this study.

## Conflict of Interest Statement

The authors declare that the research was conducted in the absence of any commercial or financial relationships that could be construed as a potential conflict of interest.
